# The Role of Snail-1 in Thyroid Cancer—What We Know So Far

**DOI:** 10.3390/jcm10112324

**Published:** 2021-05-26

**Authors:** Katarzyna Wieczorek-Szukala, Andrzej Lewinski

**Affiliations:** Department of Endocrinology and Metabolic Diseases, Medical University of Lodz, 93-338 Lodz, Poland; katarzyna.wieczorek@umed.lodz.pl

**Keywords:** thyroid cancer, Snail-1, EMT, metastasis, invasiveness

## Abstract

Thyroid carcinomas, despite the usually indolent behaviour and relatively good overall prognosis, show a high tendency to gain invasive phenotype and metastasise in some cases. However, due to a relatively slow progression, the exact mechanisms governing the metastatic process of thyroid carcinomas, including the epithelial-to-mesenchymal transition (EMT), are poorly described. One of the best-known regulators of cancer invasiveness is Snail-1—a zinc-finger transcription factor that plays a key role as an EMT inducer. More and more attention is being paid to the role of Snail with regard to thyroid cancer development. Apart from the obvious implications in the EMT process, Snail-1 plays an important role in the regulation of chemoresistance of the thyroid cells and cancer stem cell (CSC) formation, and it also interacts with miRNA specific to the thyroid gland. The aim of this review was to summarise the knowledge on Snail-1, especially in the context of thyroid oncogenesis.

## 1. Introduction

Thyroid cancer remains one of the most common malignancies of the endocrine system. Its frequency rate is gradually increasing; from 1990 to 2013, the global age-standardised incidence of thyroid cancer increased by 20% [1]. It was also found that thyroid cancer will replace colorectal cancer as the fourth leading cancer diagnosis in the United States by 2030 [2]. This global tendency may be dependent on several factors, including environmental risk factors, other chronic diseases or individual risk factors (such as obesity) or the increased detection of early-stage tumours [1].

The most common thyroid cancers, accounting for over 90% of cases, are differentiated thyroid carcinomas (DTC), deriving from the follicular cell of the gland. Within this group, papillary thyroid carcinoma (PTC) constitutes the majority of thyroid carcinoma cases (ca. 90%). The remaining cases include: follicular thyroid carcinoma (FTC), Hürthle cell carcinoma, medullary thyroid carcinoma originating from the parafollicular C cell of the gland and, rarely, anaplastic thyroid carcinoma (ATC)—the undifferentiated cancer that derives from thyroid follicular cell [3].

Most thyroid carcinomas represent biologically indolent behaviour and have a good prognosis with long-term survival. For DTC, for example, the 10-year survival rate is approximately 93% [4]. Nevertheless, around 20% of patients relapse after initial treatment, and due to its resistance to radioactive iodine, occurring metastases may not further respond to conventional therapies [4,5]. The vast majority of relapsed disease cases occur within the first ten years. Among them, more than one-fifth manifest as distant metastases, mainly in the lungs and bones [6]. Furthermore, poorly differentiated thyroid carcinoma (PDTC) and anaplastic (undifferentiated) thyroid carcinoma represent a small proportion of thyroid tumours, at the same time being the most frequent cause of death from thyroid cancer [7]. Elucidating the molecular alterations associated with the aggressive behaviour of thyroid tumours is crucial for developing novel and more effective therapeutic strategies. 

It has been described that the invasiveness of many types of cancer, including thyroid cancer, is associated with multiphase processes, such as the epithelial–mesenchymal transition (EMT). During this conversion, epithelial cells lose contact with other cells within the tissue and gain mesenchymal properties, which may lead to metastatic dissemination [8].

Snail-1, a zinc finger transcription factor, is one of the most potent EMT inducers and one of the most frequently investigated proteins related to cancer progression [9]. As previously discovered, the expression of Snail-1 correlates positively with metastasis, recurrence, tumour grade and poor prognosis in various tumours [10,11,12]. Therefore, it is critically important to understand the complexity of the molecular mechanisms that control its expression and functions.

However, relatively little research concerning the role of Snail-1 in cancer invasiveness has been performed with regard to thyroid cancer. As this type of malignancy usually presents long-term progression, its metastatic mechanisms are poorly described. Currently, the growing amount of evidence indicates that the mechanisms driving carcinogenesis may slightly differ depending on the specific type of cancer [13].

In this review, a particular emphasis was put on the previous and new reports on the role of Snail-1 in thyroid cancer progression, its aggressiveness and its mutual relationship with other factors that are important for this process.

## 2. Snail Transcription Factor Family

The Snail transcription factors superfamily plays a crucial role in multiple processes that are related to cell survival and differentiation. The members of the Snail superfamily can be divided into two related but independent groups: the Snail and the Scratch families [14]. All vertebrate Snail family members are C2H2-type zinc finger transcription factors containing the N-terminal SNAG domain.

The Snail family consists of Snail-1 (Snail), Snail-2 (Slug) and Snail-3 (Smuc), which all share an evolutionarily conserved structure. However, Snail-1 has four zinc finger structures, while Slug and Smuc each have five [14]. 

Snail-1 is a protein that is composed of 264 amino acids, with a molecular weight of 29.1 kDa. The Snail-1 protein in humans is encoded by the *SNAI1* gene, which is 2.0 kb, contains 3 exons and is located on chromosome 20q.13.2. At the transcriptional level, Snail-1 activity may be regulated via other multifunctional factors that are able to interact directly with the Snail-1 promoter, for example, HIF-1α, NF-κB, the Notch intracellular domain, IKKα, SMAD, HMGA2, Egr-1, PARP-1 or STAT3 [15,16].

Generally, Snail-1 plays the role of a transcriptional repressor that binds to regulatory regions and promoters containing sequences called E-boxes. However, as the E-box sequences are present in the promoters of a number of different genes, Snail-1 has a very wide field of activity, also serving as a regulator of key genes involved in carcinogenesis [17] (Table 1).

Snail-1’s primary function is undoubtedly the repression of E-cadherin (Table 1) but also the repression of other characteristic epithelial markers. During embryo development, such regulation (as a part of the EMT programme) is crucial for the formation of the mesoderm and the neural crest. The *SNAI1* gene is fundamental for gastrulation in the normal development of mice. It was shown that knocking out Snail-1 is lethal, as embryos seem unable to produce mesoderm, causing gastrulation failures [14]. However, in the context of cancer development, Snail-mediated EMT remains its most important and currently studied role [15,17].

Apart from the direct influence on target genes, our own research documented that Snail-1 may also influence the expression and cellular location of other intracellular proteins, such as filamin-1, which modulates cancer cells’ adhesive and migratory properties [32]. In addition, we also discovered that *SNAI1* overexpression changes cancer cells’ cytoskeleton structure by inducing TUBB3 upregulation and specific compartmentalisation in the early stages of metastasis in colon cancer cells [33].

As the role of Snail-1 in the general regulation of many physiological and pathological processes was thoroughly reviewed before [15,17,34], in this review paper, we focused on its particular role in thyroid cancer development. 

## 3. The EMT Process in the Context of Thyroid Cancer and Snail-1

**Figure 1:**
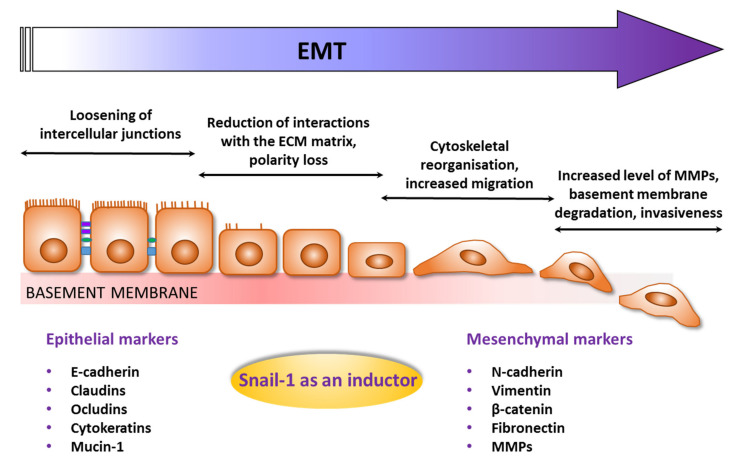
Schematic steps of the EMT process with examples of epithelial and mesenchymal markers (ECM—extracellular matrix; MMPs—matrix metalloproteinases).

The epithelial-to-mesenchymal transition (EMT) is a physiological process that occurs during early developmental stages. However, it is also characteristic of cancer progression, with it being the main mechanism responsible for invasiveness and metastasis of the neoplasm at the advanced stages [35]. During this transformation (Figure 1), the immotile cells of epithelial origin gradually lose their tight junction connections, their characteristic shape and polarity, and due to the cytoskeleton rearrangements, gain a fibroblast-like mesenchymal phenotype. The expression of characteristic markers dramatically changes. The usual epithelial markers, such as E-cadherin, cytokeratins, ocludins or mucin-1, are replaced with mesenchymal markers, like vimentin and fibronectin [27,36].

In the context of cancer progression, the most important cell ability that is enabled by the EMT is invasion. Transforming cells usually show increased activity of adhesion molecules that contribute to cell movement and stimulate proteases on the cell surface, leading to the digestion of extracellular matrix (ECM) components. The increased activity of matrix metalloproteinases (MMPs), such as MMP-2 and MMP-9, is frequently noted and may allow cells to break through the basement membrane and enter the bloodstream through intravasation [37]. In the next step, the circulating tumour cells exit the bloodstream to form micro-metastases and continue outgrowth at metastatic sites. The specific secretion of MMPs was also demonstrated in vitro in thyroid cells at the early stages of the EMT [38,39].

The EMT process in thyroid cancer may be activated by diverse factors, such as tissue hypoxia or extracellular cytokines, e.g., transforming growth factor β (TGF-β) [8]. The metabolic pathways initiated by these stimuli, including mitogen-activated protein kinase (MAPK), Wnt, Notch or phosphatidylinositol-3-kinase (PI3K), activate transcription factors, such as Snail-1, Twist or Zeb [40]. Importantly, it was also reported that such transcription factors may initiate the EMT process itself [10]. 

The induction of the EMT in the context of cancer invasion remains Snail-1′s most studied function. As it was previously mentioned, its primary mechanism is the repression of E-cadherin, which is the protein that is crucial for the formation of adherens junctions and binding cells with each other. This results in reduced cell adhesion within tissue and it promotes migratory abilities, in turn leading to reduced cell adhesion and promoting migratory phenotype [15]. By suppressing other various epithelial markers (Table 1: claudin, occludin, desmoplakin, cytokeratin and mucin-1) and upregulating mesenchymal markers (such as fibronectin and vitronectin) associated with the mesenchymal phenotype, Snail-1 serves as a key regulator of the EMT [17].

The contribution of Snail-1 in development of the metastatic thyroid cancer was also documented. Wang et al. showed that high mRNA and protein expression levels of Snail-1, TGF-β1 and MMP-9 were significantly correlated with lymph node metastases in PTC specimens [38]. 

Importantly, a significant relationship was also demonstrated between the expression of Snail-1 and the TNM thyroid cancer staging system. Mato et al. succeeded in documenting that patient samples diagnosed as stages I and II PTC showed an over 8-fold higher expression of *SNAI1* in comparison to controls, where stages III and IV were even over 15-fold higher [41]. In parallel, these observations were recently confirmed in our laboratory. Namely, we found that the overexpression of *SNAI1* indeed correlated with the metastatic phenotype in human PTC material [42].

## 4. The Interplay of BRAF^V600E^ and Snail-1 in Thyroid Cancer

Although the EMT in thyroid oncogenesis may be initiated and driven via multiple mechanisms [8], in the context of the mutual interactions with Snail-1, one particular mechanism arouses gradually more interest: the interplay between BRAF^V600E^ mutations and Snail-1. 

The B-Raf proto-oncogene (BRAF) gene is located on chromosome 7q34 and is responsible for encoding a cytoplasmic protein kinase—a part of the mitogen-activated protein kinase pathway (MAPK). BRAF oncogenic mutations lead to a permanent activation of the MAPK signaling, which in turn results in increased cell proliferation, apoptosis resistance and uncontrolled tumour growth [43]. The substitution of valine (V) by glutamate (E) at position 600 of the B-Raf protein, accounts for approximately 90% of all BRAF mutations and is designated as V600E [44]. BRAF^V600E^ mutations were identified in a number of different malignancies, including melanoma, as well as serous ovarian and colorectal cancers [45]. 

It also remains the most common alteration in PTC tumorigenesis [46]. This mutation was previously considered as a marker of the more aggressive course of the PTC [47]. Importantly, regarding in vitro ATC cell lines, it was reported that BRAF^V600E^ itself plays a key role in the specific induction of the EMT programme via changes in Snail-1 and E-cadherin protein expression levels. 

The overexpression of BRAF^V600E^ directly enhances the upregulation of Snail-1 protein; moreover, such a process significantly induces cell migration and invasion [48]. The quoted authors speculated that the activity of GSK3-β kinase could be decreased by a BRAF^V600E^ mutation, leading to subsequent proteasomal degradation of Snail-1. This regulatory mechanism may constitute the background of the BRAF^V600E^–Snail-1 interaction. Similar observations were made by Massoumi et al. in melanoma cells, where the induced hyperactivation of the BRAF^V600E^ resulted in Snail-1 overexpression, which resulted in increased cell proliferation and invasion via regulation of the CYLD tumour suppressor [49].

**Figure 2:**
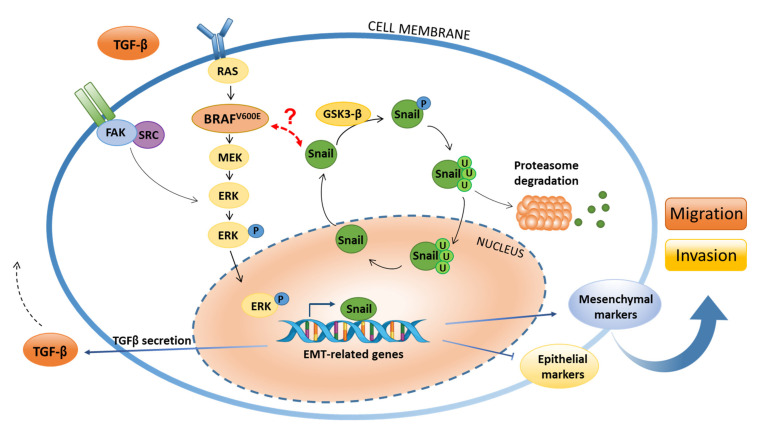
Scheme of the interactions between BRAF^V600E^ and Snail-1 that are regulated via the GSK3-β kinase in a thyroid carcinoma cell.

An additional mechanism of Snail-1 stabilisation in the cytoplasm was also demonstrated. The formation of the Snail–GSK-3β complex kinase is indeed an important regulatory element in this system and it was previously documented that specific phosphorylation of Snail-1 by the kinase ATM may also inhibit this complex formation, which prevents the degradation in the proteasome and further stabilises Snail-1 in the cytoplasm [50]. In the continuation of the ATC model studies, Baquero et al. documented that BRAF^V600E^ induces TGF-β secretion in thyroid cancer cells and that this cytokine further induces EMT cascade through the Src/FAK pathway. Nevertheless, it was shown that the activation of the Src/FAK complex by TGF-β seems independent of BRAF^V600E^ signaling and that BRAF^V600E^, TGF-β and the Src/FAK complex may act cooperatively but independently in the regulation of EMT induction and cell motility in thyroid cancer [51] (Figure 2).

A BRAF^V600E^ mutation induces the MAPK/ERK signaling pathway, leading to the induction of EMT-related gene transcription, which is also mediated via Snail-1 in the nucleus. BRAF^V600E^ also significantly induces TGF-β secretion, which in turn activates the Src/FAK complex via an autocrine loop, resulting in independent EMT stimulation. The expression of epithelial markers is downregulated, whereas mesenchymal markers are upregulated, which is reflected in the increased invasiveness and migratory properties of carcinoma cells. At the same time, Snail-1 is subjected to changes in the cellular localisation that is controlled by the GSK3-β kinase that phosphorylates Snail-1 and induces its proteasomal degradation. However, the possible mechanism of the interplay between BRAF^V600E^ mutation, GSK3-β kinase and Snail-1 remains unknown. 

However, as the conclusions drawn from in vitro studies are often difficult to apply to the human model, our own research made contributions to the studies of the Snail-1 and BRAF^V600E^ relationship in aggressive thyroid cancer human tissue. It was discovered that the expression of *SNAI1* is significantly higher in a metastatic PTC group harbouring a BRAF^V600E^ mutation, but not in metastatic BRAF^V600E^-negative PTC tissue. In patients without metastases, there were no differences in *SNAI1* expression, regardless of the BRAF^V600E^ mutation status. Moreover, these mechanisms seem to be independent of the expression of the TGF-β1 gene since no statistically significant differences were observed in metastatic and non-metastatic groups of PTC patients with BRAF^V600E^ [42].

BRAF^V600E^ does not necessarily correspond with the invasive thyroid cancer phenotype and gradually more studies indicate that it is not a good predictor of an aggressive clinical course of PTC as a marker that is tested alone [52,53]. However, our recent findings point out that increased *SNAI1* expression directly correlates with a BRAF^V600E^-positive status, especially in metastatic PTC tissue [42]. We suggested that this phenomenon may be linked to the mechanisms proposed before by Baquero et al., i.e., the GSK3β inhibition pathway [48,51], but the true nature of these interactions remains to be discovered.

The coexistence of BRAF^V600E^ and TERT promoter mutations was previously shown to increase the aggressiveness of PTC. These markers together may be regarded as a genetic diagnostic panel that is useful for establishing a poor prognosis of thyroid cancer [54].

Poma et al. recently concluded that five proteins (4E-BP1_pT70, Chk1_pS345, Snail, STAT5 alpha and PAI-1) are significantly associated with survival in different stages of PTC, and together with the TERT mutation, such a panel may refine the risk stratification and prognosis of an outcome [55]. Little is known about the mutual interactions between Snail-1 and TERT. Recently, Mazzolini et al. discovered that Snail-1 regulates TERT RNA expression and telomerase activity in mouse mesenchymal stem cells [56]. However, so far, no link between Snail-1 expression, BRAF mutations and TERT mutations has been found, as well as no data concerning the mechanisms that are common for those markers in the thyroid has been gathered.

The growing knowledge on the mechanisms regulating the interplay between Snail-1 and BRAF^V600E^ in malignant thyroid tumours casts a completely new light on metastatic thyroid cancer development and helps us to clarify the complexity of this process. A better understanding of these relations may be crucial in the development and improvement of the most promising thyroid carcinoma therapy, namely, small-molecule tyrosine kinase inhibitors (TKIs), such as Sorafenib, which is a BRAF-inhibitor that was already approved for radioiodine-resistant metastatic differentiated thyroid cancer therapy [57,58].

## 5. The Expression of Snail-1 in Thyroid Tissue

In human organisms, Snail-1 is expressed physiologically in the kidney, thyroid, adrenal gland, lungs, placenta, lymph nodes, heart, brain, liver and skeletal muscle tissues [14]. Consequently, Snail-1 is also detected in many types of cancer, such as breast, bladder, cervical, colorectal, gastric, hepatocellular, ovarian, pancreatic or prostate carcinoma [15]. Its overexpression usually correlates with increased migration, invasion and metastasis. As Snail-1 directly represses E-cadherin expression, an inverse relationship in more developed cancer stages is usually observed [59].

Moreover, Mitchell et al. compared Snail-1 expressions depending on the histologic variant of PTC. However, in this particular study, no statistically significant correlations were noted between Snail-1, E-cadherin and histopathologic prognosticators. The only trend was demonstrated between Snail expression and tumor size <5 cm (*p* = 0.07) [60]. 

In the thyroid gland, E-cadherin is commonly detected both in benign thyroid lesions and differentiated thyroid cancer. Its aberrant, reduced expression may be characteristic of thyroid cancer cell lines and invasive human PTCs [61]. Few groups have studied the relationship between Snail-1, cadherin and the progression rate in thyroid cancer samples. Hardy et al. [62] discovered that both *SNAI1* and *SNAI2* are not expressed in cells derived from normal thyroid tissue or in normal human thyroid samples, but are highly expressed in cell lines derived from thyroid carcinomas, in human thyroid carcinoma samples and their metastases. Both proteins Snail-1 and Snail-2 are also highly expressed in histological samples of follicular carcinomas of all pathological stages. There was no immunoreactivity for neither Snail-1 nor Snail-2 in morphologically normal follicles in any specimen analyzed. Moreover, these observations were also found for a specific animal model, namely, CombitTA-Snail mice, in which *SNAI1* levels were upregulated, and consequently, the mice developed PTC after the introduction of cancer cells. The authors concluded that Snail-1, which was present in minimally invasive follicular carcinomas (MI-FTC) but also overexpressed in invasive carcinoma and lymphomatous metastases, was involved in both tumour formation and dissemination during the tumour metastasising process.

Recently, the group of Wu et al. [63] examined the expression of Snail-1 protein and two different EMT regulators, namely, Slug and Twist, in human tissues of follicular thyroid tumours of different invasion phenotypes. It was demonstrated that Snail-1 expression was indeed higher in widely invasive follicular thyroid cancer (WI-FTC), PDTC and ATC groups, but decreased in follicular adenomas and MI-FTC tissues in patients [63]. 

Moreover, this research focused on the very important issue of the local expression of particular proteins related to the EMT process and cancer invasion, such as Snail-1. Invasive epithelial cancers are a kind of cancer cell assembly that are connected by stable cell–cell junctions that move through the extracellular matrix (ECM) together. The migrating cell collectives maintain a specific polarity, where a group of cells serves as the “invasive front” and the cells at the opposite end follow them [64]. As reported before, and in the studies on PTC, EMT regulators may mainly be expressed in the invasive front of the widely invasive carcinomas, which are usually associated with decreased E-cadherin expression. Wu et al. presented similar observations in the case of high-grade follicular cancer tissues, where Snail-1 overexpression was commonly observed in the invasive carcinoma fronts rather than the tumour center areas [59,63].

These results show that the immunohistochemical staining results should be interpreted with great caution, often by taking into account the entire image of the tumour, not only a fragment of it.

## 6. Snail-1 Is Implicated in the Formation of the Thyroid Cancer Stem Cells

It is generally believed that solid tumours, including thyroid carcinomas, are usually heterogeneous, both at the molecular and histological levels. Such cancer cellular heterogeneity has so far been explained by two divergent theories. The first one, called the stochastic model, assumes that cancer development is initiated by the accumulation of genetic mutations in one single cell. Consequently, random mutations within oncogenes and tumour-suppressor genes can result in uncontrolled proliferation. The second possible theory is the hierarchical model, which suggests that tumour cells originate from a small population of cells, called cancer stem cells (CSCs), that show a resemblance to normal adult stem cells [65]. This unique subset of cells exhibits exclusive self-renewal ability and metastatic and clonogenic potential. According to this model, tumour development occurs when particular CSCs are able to self-renew and differentiate giving rise to phenotypically diverse cancer cells [66]. It was described in in vitro experimental conditions that the CSC of the thyroid grow as spheres (also called thyrospheres) [67].

Previous research showed that the EMT process can induce cancer stem cell-like phenotype in a number of tumour types. Snail-1 expression, as one of the EMT regulators, may induce particular subsets of CSC-like populations, for example in breast cancer, head and neck or human squamous cell carcinoma cell lines [68,69]. With regard to the thyroid gland, a few studies have detected a significant number of resident stem cells in animal models and human thyroid tissue [70]. However, the role of thyroid cancer stem cells in cancerogenesis and further thyroid tumour progression and their link with Snail-1 transcription factor is poorly documented.

Using the model of human ATC cell lines, Yasui et al. showed that Snail-1 overexpression significantly induced (approximately 14-fold) sphere formation abilities in ALDH-cells [71]. Aldehyde dehydrogenase (ALDH), which serves as a thyroid CSC marker, appeared to show changed functioning after Snail-1 expression. The numbers of ALDH+ cells decreased while ALDH– cells gained greater sphere-forming ability than ALDH+ cells. It was concluded that while the expression of Snail-1 is frequently restricted at the invasive fronts of thyroid cancers with a characteristic EMT phenotype [72], the role of ALDH in CSC properties may be dependent on the EMT status, and Snail-mediated pathways.

Interestingly, Heiden et al. reported that the sonic hedgehog molecular pathway (Shh) plays an important role in maintaining CSC self-renewal in ATC cells [68]. The expression of Snail-1 is regulated via the Shh pathway and is correlated with the expression of ALDH and an increased ALDH^High^ CSC population. Furthermore, the knockdown of *SNAI1* by siRNA consequently results in a decreased number of ALDH^High^ thyroid CSCs in cell line models [68].

In turn, using a murine thyroid papillary BRAF^V600E^ positive carcinoma model, Ma et al. indicated that cells overexpressing Snail-1 show an increased level of specific stem cell markers, such as Oct4, Rex1 and CD15. It also appears this CSC-like phenotype directly stimulates the migratory potential of the cells [70]. The above studies led to a very important conclusion: the PTC cells induced by a BRAF^V600E^ mutation in mice undergo EMT, and through Snail-dependent pathways, dedifferentiate to acquire stem-cell-like features. These data support the stochastic model of CSC origin, suggesting that every malignant thyroid cancer cell has the potential to act as a CSC.

Finally, CSC in thyroid cancer may be dependent on the external environment in the thyroid gland, for example, components of the inflammatory process. Chronic thyroid inflammation plays an important role in the pathogenesis of thyroid cancer [73]. The very recent studies of Zheng and colleagues showed a new relationship between Snail-1 and IL-6 in thyroid CSC derived from ATC cell lines [74]. After a 3-day IL-6 exposure of the in vitro CSC culture, the expression of Snail-1 was significantly increased. IL-6 also promoted a colony-forming ability. Nevertheless, although authors suggested that the proliferative effect of thyroid cancer stem cells depends on the activation of the IL6/JAK1/STAT3 pathway, the specific role of Snail-1 in this process has not been further clarified.

## 7. Snail-1 Induces Chemoresistance in Thyroid Cancer

The gold-standard treatment for thyroid carcinoma, in general, is surgery combined with radioactive iodine therapy [75]. 

However, chemotherapy is rarely used in some cases of aggressive, advanced thyroid carcinomas; in conjunction with radiation therapy, it may be applied in order to stop the metastases from spread to other parts of the organism [76]. 

The most commonly used drugs are taxanes (such as paclitaxel or docetaxel), anthracyclines (usually doxorubicin) or platinum compounds (mostly cisplatin) [77]). Although the efficacy of initial chemotherapy is usually high, the response rate may significantly decrease with the length of treatment. The reason for this is the acquisition of chemoresistance to xenobiotics by cancer cells. The molecular mechanisms involved in the development of chemoresistance are complex, but its main important factors are specific membrane protein transporters, belonging to the ATP-binding cassette (ABC) superfamily, that are able to efflux anticancer agents outside the cell [78]. Commonly expressed on many cells of various cancers, such as breast, colon or prostate cancer, ABC transporters were also detected in the murine thyroid gland [79] and a large number of thyroid cell lines [80].

Generally, the most frequently expressed form of ABC transporter implicated in cancer drug resistance is ABCB1; other types of ABC proteins are less common. However, it was previously reported that in thyroid cancer (mostly ATC), ABCG2 and ABCC1 proteins seem to be the most highly expressed transporters [81]. A few studies also demonstrated that ABC transporters are highly expressed by CSCs of ATC, making them potential markers of the thyroid cancer invasive phenotype [81,82]. Later studies by Yasui et al. suggested that ATC cells not only show the CSC phenotype but also they are more resistant to 5-FU, a drug that is commonly used in cancer chemotherapies [71]. Nevertheless, the more precise role of ABC proteins in this model remains unknown. 

Interestingly, it appears that the mechanisms of ABC proteins may also be regulated by the Snail-1 factor. Mato et al. documented, for the first time, that the high increase in expression of the ABCG2 correlates with the overexpression of *SNAI1* and two other EMT-related transcription factors, namely, Twist1 and ZEB1 genes in PDTC, ATC and PTC tissues in patients [41]. The findings also correlated with more aggressive disease stages accordingly to the TNM scale, where gradually higher ABCG2 and *SNAI1* expression levels were noted at stages III and IV [41]. These results may suggest that ABCG2 and Snail-1 together play a crucial role in thyroid tumour cell dedifferentiation when acquiring an invasive phenotype.

However, as the majority of the studies concerning multidrug resistance in thyroid cancer were conducted on cell lines in vitro, more research is required to fully understand the complex mechanisms of chemoresistance in human models.

So far, the most potent therapy that is currently recommended by NCCN (National Comprehensive Cancer Network) is the usage of a wide number of TKIs, such as Suntinib, Axitinib, Levantinib, Vandetinib or Sorafenib. As a systemic therapy, these compounds were successfully used in advanced or metastatic PTC, FTC, ATC and other sporadic cases of thyroid cancer [57,58]. To date, little is known about whether Snail-1 may contribute to the development of resistance to TKIs in the thyroid. Nevertheless, there are already reports that Snail-1 may induce resistance to Gefitinib in non-small cell lung cancer [83].

## 8. miRNA and Snail-1 in Thyroid Cancer

MicroRNAs (miRNAs) are short, single-stranded RNAs that may modulate gene expression at the post-transcriptional level. They are able to bind to the 3′-untranslated region (3′-UTR) of target mRNAs, which leads to the degradation or inhibition of their translation. A large amount of evidence suggests that miRNAs are involved in multiple physiological processes, including differentiation, immune system regulations, cell proliferation, migration or invasion. Importantly, miRNAs also play a crucial role in the pathogenesis and progression of various tumours, functioning as either oncogenes or suppressors depending on the roles of their target genes [84].

In vitro and in vivo experiments have revealed the role of several miRNAs in the pathogenesis of thyroid cancer [85]. For example, in the study conducted by Rosignolo et al., miR-146a-5p and miR-221-3p were chosen from 754 other miRNAs as the most promising biomarkers, whose expressions were significantly increased in patients with PTC prior to thyroidectomy [86].

The influences of the expression levels of miR-146a and miR-146b on thyroid cell proliferation and migration via DICER1 or TLRs/IL-1 pathways were previously found by different scientific groups [87]. Furthermore, miR-221 was shown to stimulate PTC K1 cell proliferation, migration and invasion, possibly acting via the TIMP3 molecular pathway [88]. 

The role of numerous particular miRNAs in thyroid cancer development was postulated [85]. However, although miRNAs regulate the function of metabolic pathways that are closely linked to cancer progression, such as PI3K/Akt/mTOR, MAPK, Wnt or NF-κβ, only a few sources have demonstrated the direct link between the Snail-1 transcription factor and particular miRNAs.

Recently, Ma et al. discovered that miR-199a-5p inhibits the progression of PTC by targeting *SNAI1* [89]. The expression of this miRNA was found to be downregulated in other different tumors, such as breast cancer, colorectal cancer, hepatocellular carcinoma and in PTC tissues compared with normal tissues [85,90]. Such an elevated expression of miR-199a-5p was confirmed in specific, stably transfected PTC K1 cell lines as well [89]. Interestingly, authors showed that restoring miR-199a-5p expression significantly slowed cell migration, invasion and the EMT process in PTC cells, while its downregulation caused the reverse effect [89]. 

At the same time, it appears that Snail-1 is highly expressed in the studied PTC tissues and cells in vitro. The Target Scan analysis, which is a modern method seeking potential candidate genes for particular miRNAs, identified *SNAI1* as a potential target of miR-199a-5p. Further experiments, such as the luciferase reporter assay and Western blot, demonstrated that miR-199a-5p suppresses SNAI1 expression by directly targeting its 3′UTR region [91]. These findings were also confirmed in animal xenograft tumour model models in vivo. Animals with injected cells that were overexpressing miR-199a-5p showed slower tumour growth rates and lower volumes and tumour weights than in the controls [89]. More interestingly, it appears that miR-199a-5p overexpression in xenografts suppresses the tumour growth of PTC by downregulating *SNAI1* [89].

Han et al. observed the close link between Snail-1 and miRNA-215 [92]. miR-215 is a tumour suppressor in renal cell carcinoma, gastric cancer, glioma and colorectal cancer [93], which is significantly downregulated in PTC tissues and cell lines. miR-215’s lower expression was associated with PTC tumour size, metastasis status and differentiation. Importantly, it was shown that the overexpression of miR-215 significantly suppresses the tumour proliferation and metastasis of PTC by directly targeting the ADP ribosylation factor guanine nucleotide-exchange factor 1 (ARFGEF1). Further analyses revealed that miR-215 overexpression or ARFGEF1 knockdown inhibited EMT, while miR-215 silencing and ARFGEF1 ectopic expression promoted it.

The AKT/GSK-3β/Snail axis is an important pathway that takes part in modulating the EMT process in tumours. miR-215 can inhibit AKT phosphorylation that is mediated by ARFGEF1, and subsequently, impair GSK-3β phosphorylation and Snail expression in PTC cells [93]. The above findings led to the general conclusion that miR-215 inhibits EMT via ARFGEF1-activated AKT/GSK-3β/Snail signaling.

Another miRNA that seems to be associated with the expression of Snail-1 is miR-150-5p. The impact of this miRNA on tumour progression was previously documented in other cancer malignancies, for example, cholangiocarcinoma or gastric cancer [94]. Studies performed in vitro on human PTC cell lines with a BRAF^V600E^ oncogene revealed that the overexpression of miR-150-5p results in a decrease in E-cadherin expression but an enhancement of mesenchymal markers expression, such as N-cadherin, Slug, vimentin, ZEB1 and Snail-1 [95]. The observations of Yan et al. led to the conclusion that miR-150-5p may enhance the EMT process through the MEK/ERK signalling pathway and control the BRAF^V600E^ oncogene.

So far, the three aformentioned studies are the only ones that show direct interactions between specific miRNA and Snail-1 in thyroid cancer. However, taking into account the fact that miRNAs regulate so many different molecular pathways that are involved in carcinogenesis and cancer progression, it seems only a matter of time and development of more accurate techniques that the link between Snail-regulated pathways and more miRNA candidates playing a vital role in PTC development will be discovered.

The most important facts on the role of Snail-1 in thyroid cancerogenesis are gathered in Table 2.

## 9. Conclusions

Undoubtedly, Snail-1 is one of the most important regulators that are involved in the development of cancer and its invasion. In thyroid carcinomas, probably due to the long-termed development of metastases, the interactions of Snail-1 remain rather poorly investigated in comparison to other cancers, such as breast or lung tumours. However, it appears that the association and related mechanisms of action between Snail-1 and some target proteins or genes that are unique to the thyroid gland may underlie the regulation of tissue-specific carcinogenesis mechanisms.

One of these important interactions occurs between Snail-1 and BRAF^V600E^, potentially co-regulating migration abilities and the traits of the invasive phenotype of thyroid cancer cells. Furthermore, the interplay between Snail-1 and specific miRNAs, in particular, miR-199a-5p, miRNA-215 and miR-150-5p, seems to function as a complex regulatory system of the EMT in thyroid carcinomas. Similarly, as in other carcinomas, Snail-1 may induce thyroid cancer dedifferentiation and CSCs formation, which is also linked with the acquisition of a chemoresistant cell phenotype. 

It was recently suggested that in low- or intermediate-risk DTC, lobectomy or total thyroidectomy seem sufficient treatment and the usefulness of radioiodine is being questioned [96].

Since Snail-1 remains a promising factor that could be implemented as a part of a molecular diagnostic panel for thyroid cancer; the evaluation of its expression may also be effective and useful in the stratification and management of DTC cases that were not submitted to radioiodine treatment.

Summarising all the information cited above on Snail-1, one must come to an obvious conclusion: the broader the knowledge concerning the mutual interactions of Snail-1 and its target partners within the thyroid, more significant progress in the development of effective and less-invasive treatments for thyroid carcinomas was recorded.

## Figures and Tables

**Table 1:** Examples of the most important target genes that are associated with carcinogenesis regulated by Snail-1 [15].

Target Gene	Function	The Role of Snail-1	References
E-cadherin	Transmembrane glycoprotein responsible for cell-to-cell adhesion, maintains anti-migratory properties of epithelial cells	Gene repression	[18,19]
Occludin	Integral membrane protein, specifically expressed in the tight junctions in epithelial and endothelial cells	Gene repression	[20]
Claudins	Integral membrane proteins, components of the tight junctions	Gene repression	[21]
Mucin-1	Transmembrane glycoprotein, plays a protective function by binding to pathogens and as a component of cellular signaling, epithelial marker	Gene repression	[22]
RKIP	Inhibitory protein, suppresses metastasis by inhibiting the Raf–MEK–ERK and NF-κβ pathways	Gene repression	[23]
PTEN	Inhibits the PI3K pathway and serves as a tumour suppressor	Gene repression	[24]
ZEB1	Transcription factor, associated with the EMT induction, represses E-cadherin and Mucin-1	Gene repression	[22,25]
Cytokeratin 18	Intermediate filament protein associated with cell structure, signaling and cell cycle regulation; epithelial marker	Gene repression	[22]
p53	Tumour suppressor; regulates cell cycle, apoptosis and genomic stability	Gene repression	[26]
Vimentin	Intermediate filament protein; builds the cytoskeleton, giving cell flexibility and anchoring the position of organelles; mesenchymal marker	Gene upregulation	[27]
Fibronectin	Glycoprotein involved in cell adhesion, growth, differentiation and migration	Gene upregulation	[28]
LEF-1	Forms complexes with β-catenin and represses E-cadherin, mesenchymal marker	Gene upregulation	[29]
MMP-2; MMP-9	Degrades the extracellular matrix proteins, cleaves cell surface receptors and modifies cell-matrix adhesive properties	Gene upregulation	[28,30,31]

RKIP—Raf kinase inhibitor protein; PTEN—phosphatase and tensin homolog deleted in chromosome 10; PI3K—phosphatidylinositol 3-kinase; ZEB1—zinc finger E-box-binding homeobox 1; LEF-1—lymphoid enhancer-binding factor 1; MMP-2, MMP-9—matrix metalloproteinases 2 and 9.

**Table 2:** Tabular overview of the most important information on the role of Snail-1 in cancerogenesis in thyroid glands.

	References
**The EMT process in the context of thyroid cancer and Snail-1**	
Expression of Snail-1, TGF-β1 and MMP-9 is significantly correlated with lymph node metastases in PTC	Wang et al., 2014 [38].
Snail-1 expression is proportional to the advanced stages of PTC	Mato et al., 2014 [41]
**The interplay of BRAF^V600E^ and Snail-1 in thyroid cancer**	
Overexpression of BRAF^V600E^ enhances the upregulation of the Snail-1 protein and induces cell migration and invasion	Baquero et al., 2013 [48]
BRAF^V600E^ induces TGF-β secretion in thyroid cancer cells; BRAF^V600E^, TGF-β and the Src/FAK complex cooperate in the regulation of EMT induction and cell motility	Baquero et al., 2016 [51]
SNAI1’s increased expression directly correlates with a BRAF^V600E^-positive status, especially in metastatic PTC tissue	Wieczorek-Szukala et al., 2020 [42]
**The expression of Snail-1 in thyroid tissue**	
Snail-1 is overexpressed in cell lines derived from thyroid carcinomas, in human thyroid carcinoma samples and metastases; Snail-1 is involved in tumour formation and dissemination during the metastasising process	Hardy et al., 2007 [62]
Snail-1 expression is higher in WI-FTC, PDTC and ATC, but decreased in follicular adenomas and MI-FTC tissues	Wu et al., 2019 [63]
Snail-1 overexpression occurs in the invasive fronts rather than tumour center areas of high-grade follicular cancer	Wu et al., 2019 [63], Heiden et al., 2014 [68]
**Snail-1 is implicated in the formation of thyroid cancer stem cells**	
Snail-1 overexpression induces sphere formation in ALDH—cells	Yasui et al., 2013 [71].
Shh pathway plays a key role in maintaining the CSC self-renewal in ATC cells	Heiden et al., 2014 [68]
Murine BRAF^V600E^-positive PTC cells with Snail-1 overexpression gain a CSC-like phenotype and migratory potential	Ma et al., 2014 [70]
IL-6 stimulates Snail-1 expression in thyroid CSC derived from ATC cell lines [74]	Zheng et al., 2019 [74]
**Snail-1 induces chemoresistance in thyroid cancer**	
High expression of ABCG2 correlates with the overexpression of Snail-1, Twist1 and ZEB1 genes in PDTC, ATC and PTC tissues	Mato et al., 2014 [41]
**miRNA and Snail-1 in thyroid cancer**	
miR-199a-5p inhibits PTC progression by targeting SNAI1; its overexpression reduces cell migration, invasion, the EMT process and PTC tumour growth in xenografts	Ma et al., 2018 [89]
miR-215 inhibits EMT via the ARFGEF1-activated AKT/GSK-3β/Snail signaling in PTC cells and tissue	Han et al., 2019 [92]
The overexpression of miR-150-5p induces the expression of mesenchymal markers, including Snail-1, in PTC cell lines with BRAF^V600E^	Yan et al., 2018 [95]

## Abbreviations

ALDH	Aldehyde dehydrogenase
ARFGEF1	ADP ribosylation factor guanine nucleotide-exchange factor 1
ATC	Anaplastic thyroid carcinoma
CSC	Cancer stem cells
DTC	Differentiated thyroid carcinoma
EMT	Epithelial to mesenchymal transition
FTC	Follicular thyroid carcinoma
LEF-1	Lymphoid enhancer-binding factor 1
MAPK	Mitogen-activated protein kinase
MMP	Matrix metalloproteinase
PDTC	Poorly differentiated thyroid carcinoma
PI3K	Phosphatidylinositol 3-kinase
PTC	Papillary thyroid carcinoma
PTEN	Phosphatase and tensin homolog deleted in chromosome 10
RKIP	Raf kinase inhibitor protein
RTK	Receptor tyrosine kinases
TKIs	Tyrosine kinase inhibitors
TUBB3	Tubulin–3
ZEB1	Zinc finger E-box-binding homeobox 1
